# Detection of Chewing Strokes from Jaw Movement Signals in Dairy Cows Using a Nose-Mounted Accelerometer

**DOI:** 10.3390/s26134148

**Published:** 2026-07-01

**Authors:** Saskia Strutzke, Daniel Fiske, Gundula Hoffmann

**Affiliations:** 1 Department Sensors and Modelling, Leibniz Institute for Agricultural Engineering and Bioeconomy (ATB), 14469 Potsdam, Germany; 2 Gouna GmbH, 14621 Schönwalde-Glien, Germany; daniel.fiske@gouna.tech

**Keywords:** accelerometer, wearable sensor, precision livestock farming, rumination monitoring, livestock monitoring, ingestive behavior, behavior analysis

## Abstract

This study evaluated a non-invasive nose-mounted accelerometer for automated detection of chewing strokes in dairy cows. Data were collected from 15 Holstein Friesians and validated against manual video annotations. Chewing strokes were identified using a peak detection algorithm applied to smoothed acceleration data. Two algorithm versions were analyzed: a raw version and a cleaned version that excluded a five-second interval during regurgitation, where no physiological chewing occurs. The cleaned version showed higher agreement with the reference method (Intraclass Correlation Coefficient [ICC] = 0.91; 95% Confidence Interval [CI]: 0.77–0.96) and lower error metrics (Mean Absolute Error [MAE]: 3.67; Root Mean Square Error [RMSE]: 4.72; Mean Absolute Percentage Error [MAPE]: 5.64%) compared to the raw version (ICC = 0.67; MAE: 10.00; RMSE: 11.48; MAPE: 15.27%). Both methods demonstrated that reliable detection of chewing activity is feasible using this sensor system. Automated chewing stroke detection may contribute to the assessment of rumen function, feeding behaviour, and animal welfare and may support future precision livestock farming applications by providing objective information on chewing activity.

## 1. Introduction

The counting of chewing strokes in dairy cows has become an important indicator in both scientific research and agricultural practice for monitoring animal health, welfare, and metabolic activity. Chewing strokes serve as a direct metric of feed intake and rumination behavior, which are closely associated with rumen health and overall well-being. Changes in the frequency of chewing strokes have been linked to stress, illness, or feeding-related issues, making them a valuable early-warning parameter in herd management and a subject of ongoing research. Additionally, chewing activity is strongly correlated with feed structure and quality, offering insights into ration optimization [1,2,3,4].

Chewing predominantly occurs during rumination, typically 30 to 60 min after feed ingestion. During this phase, regurgitated boluses are rechewed, further crushed, and mixed with saliva, facilitating both particle size reduction and microbial colonization in the rumen (for further details see [5]). These processes are essential for effective passage of digesta into the lower gastrointestinal tract [1,4]. Salivation during chewing also plays a critical role in maintaining ruminal homeostasis, particularly by buffering rumen pH and preventing acidosis [4]. Consequently, prolonged chewing activity serves as a robust indicator of optimal rumen function [6,7]. The number of chewing strokes provides meaningful insights into both feed composition and digestive efficiency [3,8,9].

The total daily duration of rumination depends on season, species, and diet and ranges from 276 to 624 min per day in adult cows [10], distributed across approximately 10 to 21 rumination periods. Each period typically lasts 30 to 60 min, during which 30 to 60 boluses are processed. The chewing time per bolus ranges from 30 to 60 s [11], resulting in an estimated 20,000 to 30,000 chewing movements per day [1]. Stressors and variations in feed composition are known to influence chewing behavior. For instance, Ji et al. [12] demonstrated that a 1 °C increase in daily ambient temperature reduces rumination time by approximately 5.12 min. Similarly, health-related stressors, such as severe inflammatory diseases during the peripartum period, have been associated with delayed increases in rumination time postpartum, with over 90% of affected cows suffering from clinical illness [13].

Additionally, hormonal and reproductive states can impact chewing activity. Pahl et al. [14] have revealed a decline in rumination time on the day prior to and the day of insemination, suggesting its potential utility in estrus prediction.

The accurate recording of rumination activity, including chewing movements, is invaluable for advancing cattle health monitoring. Although visual counting methods are possible, they are labor-intensive, time-consuming, and prone to observer bias. Automated systems have thus become increasingly important, particularly for grazing systems [15]. For example, the RumiWatch (ITIN + HOCH GmbH, Liestal, Switzerland) system employs a pressure sensor integrated into a halter to detect chewing movements [16]. Another device, DairyCheck (research prototype), uses electrodes and acceleration sensors for the same purpose but requires further development before becoming a practical tool for herd management [17]. Innovative approaches, such as acoustic monitoring methods and single-axis accelerometers, have also been explored for detecting chewing stroke movements [18]. Some studies have implemented pressure sensors [19], strain gauges [20] and microphones [21,22] to monitor chewing activity. Recent reviews have highlighted the growing importance of accelerometer-based monitoring systems in precision livestock farming and emphasized that data preprocessing, sensor placement, and signal-processing strategies remain critical factors for reliable behavior detection, even as machine-learning and deep-learning approaches continue to emerge [23].

This study constitutes an extended validation of a system previously used and validated in research to record respiratory rate in dairy cattle [24], developed by Gouna GmbH, Germany. This study builds upon previous work demonstrating that the “GoClip” device can accurately monitor both rumination behaviour [5] and respiratory rate [24]. Since chewing activity is closely linked to rumen function, feed utilization, and animal welfare, its automated monitoring may provide valuable information for herd health assessment and precision livestock farming applications [2,3]. In addition, automated monitoring of chewing activity may provide information relevant to forage intake assessment and feeding behaviour evaluation.

Therefore, the objective of this study was to evaluate the ability of a nose-mounted accelerometer to automatically detect chewing strokes in dairy cows from jaw movement signals and to compare its performance with visual observations as the reference method. It was hypothesized that acceleration data obtained from a nose-mounted sensor can reliably quantify chewing stroke movements in dairy cows.

## 2. Materials and Methods

### 2.1. Animal and Housing

The study was reviewed and approved by the competent authority for licensing and notification procedures for animal experiments (LAVG) in Brandenburg, Germany (AZ: 2340–8–2021). The trial was conducted at the Educational and Experimental Center for Animal Breeding and Husbandry (LVAT, Groß Kreutz, Germany) in September 2024. A total of 15 Holstein Friesian cows were included. Data collection took place on a single day between 0900 h and 1500 h. The lactating cows were housed in a free-stall barn equipped with 53 lying cubicles, each bedded with a straw-lime mixture. The herd consisted of 55 animals on the day of the trial. The cows varied in lactation number (2nd to 5th lactation) and stage of lactation (between 36 and 401 days in milk), ensuring that the method was tested under different animal-specific conditions. During the trial, the animals were restrained at the feeding fence and a total mixed ration (TMR) was offered ad libitum. At the beginning of the rumination phase, the sensor device (GoClip; Gouna GmbH, Schönwalde-Glien, Germany) was attached intranasally to the nasal septum and remained in place for approximately five minutes. After the recording period, the device was removed, and the animals were released. Feed remained freely available throughout the trial period.

### 2.2. Sensor Device

**Figure 1:**
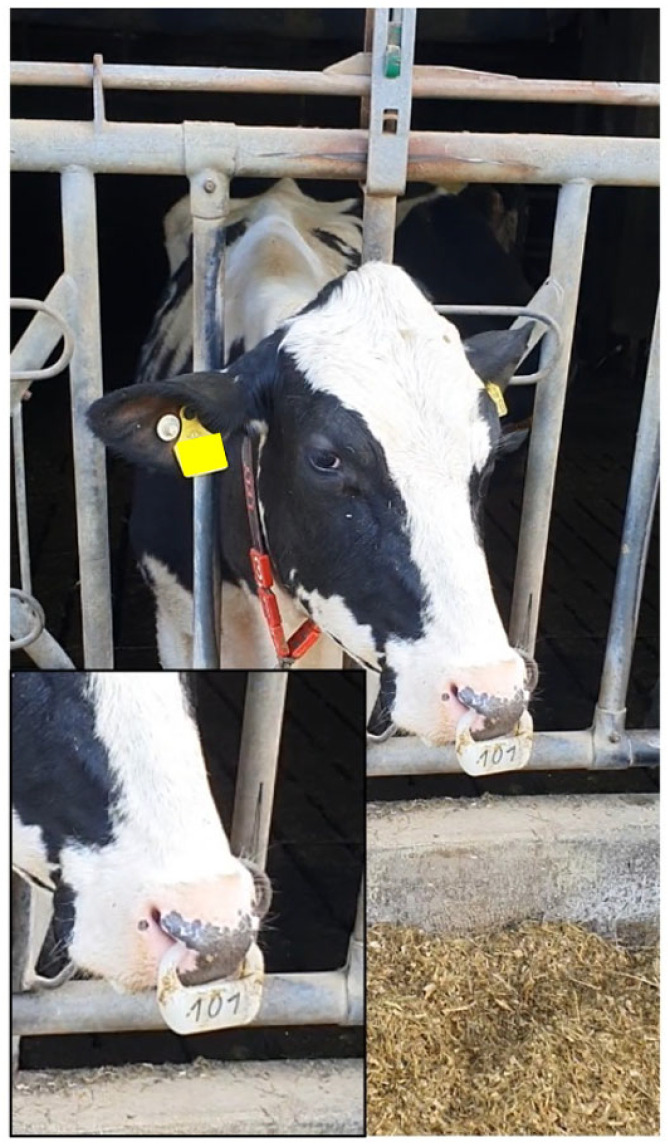
Experimental cow equipped with a sensor (GoClip, Gouna GmbH) attached non-invasively inside the nostril for detecting chewing activity during the rumination phase.

For the present study, we used the non-invasive respiratory monitoring device GoClip (Gouna GmbH, Schönwalde-Glien, Germany), which is validated [24] and already well established in both research and practical applications. The device was specifically developed for long-term studies and enables continuous and automatic recording of raw data on inhalation, exhalation, and general activity in dairy cows. The system was attached to the cows nose and was non-invasively secured inside the nostrils using retaining clips (Figure 1). The integrated chewing stroke monitor contained an acceleration sensor, a microcontroller, and a battery (total weight: 72 g, width: 9.5 cm, height: 7.0 cm).

### 2.3. Sensor Data

Continuous data acquisition was performed for five minutes per test animal. The chewing stroke monitor logged data continuously throughout the measurement period. Initially, the animals were positioned at the feeding fence with fixing option. Once the rumination phase began, the device was attached to the nose. The cows had no prior experience with the GoClip device. Therefore, a two-minute adaptation period was allowed before the start of the analyzed recording period to minimize potential effects of initial reactions to the sensor, as animals resumed normal rumination behaviour shortly after sensor attachment in previous studies using the same device. Data recording commenced immediately thereafter. The integrated 3-axis acceleration sensor captured high-resolution data at 10-millisecond intervals. A microcontroller served as the interface, retrieving sensor data and transmitting it wirelessly in real time to a nearby base station. This base station was connected to a laptop via cable, where the data were stored and made available for further analysis.

### 2.4. Visual Observations

As a reference method for data acquisition, video recordings of the head during chewing were made using a Samsung Galaxy S10 (Samsung Electronics, Seoul, Republic of Korea). Recordings were only performed when the cow remained calm and was not directly affected by external disturbances such as noise, movement within the barn, or feeding activity, in order to capture physiological chewing movements with minimal interference. In total, 15 video recordings of 5 min each were taken to ensure the capture of at least two rumination events per animal. A total of 75 min of video footage was recorded. Only segments containing clearly identifiable rumination events were included in the analysis. The final dataset comprised 30 rumination events with a combined duration of 26 min 38 s and served as the basis for the validation of the sensor-based chewing stroke detection algorithm against visual observations. The beginning and end of each chewing sequence were defined by the occurrence of a regurgitation. The videos were stored individually for each animal. Replay segments were matched to the corresponding video sequences using the real-time clock embedded in the controller in combination with the recorded video timestamps.

### 2.5. Data Processing

The generated acceleration data were stored and visualized using Python (version 3.11; Python Software Foundation, Wilmington, DE, USA). Data segmentation was performed manually based on synchronized video recordings (Samsung Galaxy S10; Samsung Electronics Co., Ltd., Seoul, Republic of Korea). Each segment corresponded to a single rumination event, defined as the period between two consecutive regurgitation events. Consequently, segment lengths were variable and depended on the duration of the respective rumination event. The start and end points of each segment were identified visually from the video recordings and matched to the corresponding sensor data using synchronized timestamps. For the cleaned algorithm, the first five seconds following each regurgitation event were excluded from further analysis.

After segmentation of the raw data, smoothing was applied to reduce noise and enhance the distinguishability of movement patterns. The sensor records acceleration signals in the nasal region. These signals are associated with jaw movements during rumination and were used as an indirect measure of chewing activity in the present study. Although the sensor records triaxial acceleration data, chewing-stroke detection in the present study was performed exclusively using the *X*-axis acceleration signal. Preliminary analyses and previous studies using the same sensor system demonstrated that the *X*-axis provided the clearest chewing-related signal patterns and the highest signal-to-noise ratio for jaw movements transmitted through the nasal region. In the present study, chewing strokes were defined as repetitive jaw movements occurring during rumination and were identified using a peak detection algorithm applied to the processed acceleration data. A moving average filter was applied first, followed by additional smoothing using a Savitzky–Golay filter (window length = 15 samples, polynomial order = 3) for more precise adjustment of the signal. The filter settings were selected empirically during preliminary analyses to achieve effective noise reduction while preserving the characteristic peak structure associated with chewing activity. The smoothed data were analyzed for the occurrence of peaks, and only the number of detected peaks was used to estimate the number of chewing strokes. Local maxima in the acceleration signal were identified using the “find_peaks” from the SciPy (Scientific Python, version 1.14.1) library. Peaks were counted during the chewing phase within each rumination event defined as the time period between two consecutive regurgitations. The number of detected peaks was used to estimate chewing frequency, which was then extrapolated to chews per minute (CPM). For further analysis, two versions of the algorithm were considered: an unfiltered version (“raw”) and a cleaned version (“cleaned”). The raw version counted all detected acceleration peaks within each defined sequence. In contrast, the cleaned version took into account that no chewing activity occurs physiologically during a regurgitation. Therefore, a five-second interval following each regurgitation was excluded from the counting. The five-second exclusion interval was selected based on observations from previous studies using the same sensor system and was confirmed by the synchronized video recordings evaluated in the present study. These observations consistently showed a pause of approximately 4–5 s between regurgitation and the resumption of rhythmic chewing activity. After this interval, detected chewing activity was again included in the analysis. Both algorithm versions were evaluated separately in the statistical analysis.

**Figure 2:**
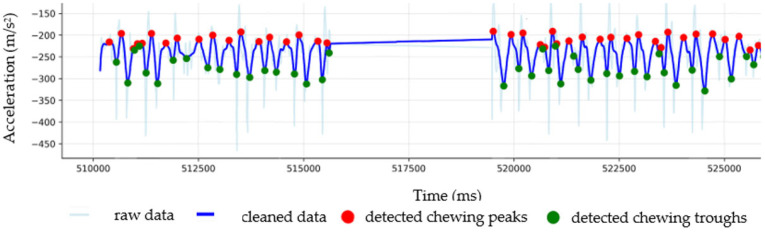
Example of accelerometer-based chewing stroke detection within a rumination segment. The picture shows the raw (light blue) and cleaned (dark blue) acceleration signal of the *X*-axis, with detected chewing peaks (red) and troughs (green).

The processed acceleration data were exported as CSV files to enable detailed analysis and serve as a basis for further investigations. An example of the accelerometer-based data processing, including signal smoothing and peak detection for chewing stroke identification, is shown in Figure 2.

### 2.6. Statistical Analysis

Data analysis was performed using Python with the pingouin statistics package (version 0.5.4) and Microsoft Excel 365 (Microsoft Corp., Redmond, WA, USA). To assess the agreement between automatically detected chewing events (sensor based) and the manual video analysis (gold standard), several statistical methods were applied. A boxplot was created to visualize the distribution of chewing counts per sequence across the three methods. It displays the median, interquartile range (IQR), whiskers (1.5× IQR), and the arithmetic mean as an additional marker. Potential outliers were identified using the 1.5× IQR rule. The accuracy of the two algorithm versions was evaluated using three error metrics: Mean Absolute Error (MAE), Root Mean Squared Error (RMSE) and Mean Absolute Percentage Error (MAPE). To statistically assess agreement between the manual and algorithm-based methods, the Intraclass Correlation Coefficient (ICC) was calculated using the ICC(2,1) model (two-way random effects, single measures). The ICC was computed for each algorithm version in comparison to the manual video analysis, which served as the reference standard. In addition, Bland–Altman plots [25] were generated to visualize systematic differences between methods. These plots show the difference between two methods against their mean, including the 95% limits of agreement.

## 3. Results

### 3.1. Descriptive Statistics and Distribution of Chewing Events

**Figure 3:**
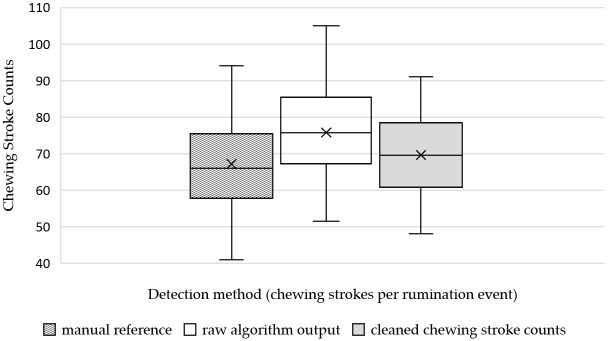
Boxplot showing the number of chewing strokes per rumination event, as detected by two accelerometer-based algorithms (raw and cleaned) and compared to the manual video annotation (reference). The X marks the arithmetic mean.

The boxplot (Figure 3) indicated a largely symmetric distribution of chewing counts across all three methods. The visual counting using video recordings yielded a median of 66.0 chewing strokes per rumination event (min: 41.0, max: 94.0) and a mean duration per rumination event of 53.3 s. The raw algorithm showed a higher median of 76.0 (min: 52.0, max: 105.0), while the cleaned algorithm resulted in a median of 69.5 (min: 48.0, max: 91.0).

The median and mean values were closely aligned and the interquartile ranges were similar, suggesting a consistent spread across datasets. Based on the 1.5× IQR rule, no statistical outliers were identified.

### 3.2. Error Metrics and Algorithm Accuracy

The error metrics demonstrated a clear improvement in accuracy for the cleaned algorithm. It achieved an MAE of 3.67, an RMSE of 4.72 and an MAPE of 5.64%, compared to the raw algorithm, which showed an MAE of 10.00, RMSE of 11.48 and MAPE of 15.27%.

### 3.3. Method Agreement and Validation

The Intraclass Correlation Coefficient (ICC) further confirmed the pattern observed in the error metrics:

- The agreement between the manual video analysis and the cleaned algorithm was excellent, with an ICC(2,1) of 0.914 and a 95% confidence interval of 0.77 to 0.96.
- The comparison between manual and raw algorithm output showed moderate agreement, with ICC = 0.667 and a 95% CI of −0.02 to 0.88.
- Between the raw and cleaned algorithm, the method agreement was good, with ICC = 0.765 and a 95% CI of 0.13 to 0.92.

**Figure 4:**
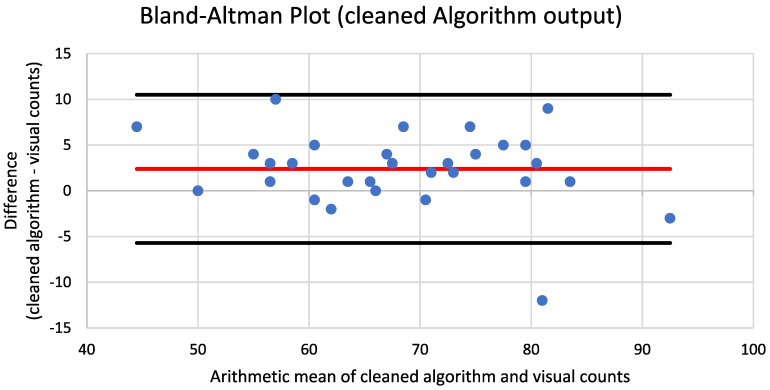
Bland–Altman Plot comparing chewing count estimates between the cleaned algorithm and manual video analysis. The plot shows the difference between both methods against their mean. The red line indicates the mean bias (2.4), and black lines represent the 95% limits of agreement (−5.70 to 10.50). Blue dots represent individual observations.

**Figure 5:**
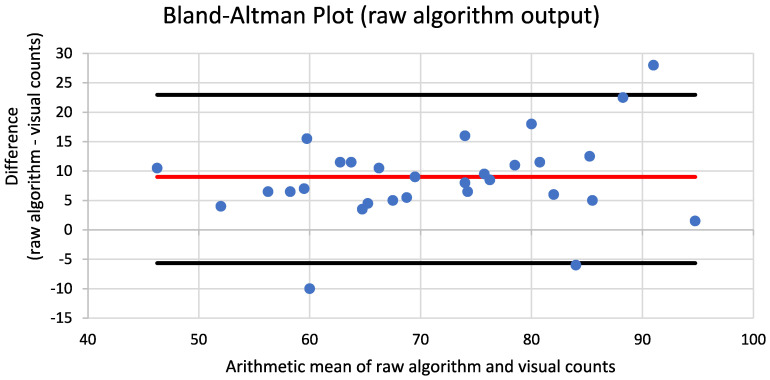
Blue dots represent individual observations. The red line indicates the mean bias (8.93), and the black lines represent the 95% limits of agreement (−5.44 to 23.31).

The Bland–Altman analysis highlighted the difference in accuracy between the two algorithm versions (Figure 4 and Figure 5). The cleaned algorithm exhibited a low mean bias of 2.4 chewing events and narrow 95% limits of agreement (−5.70 to 10.50), whereas the raw algorithm showed a considerably higher bias (8.93) and much wider agreement limits (−5.44 to 23.31). These findings emphasize the improvement in measurement consistency and agreement achieved through data cleaning.

## 4. Discussion

The system presented in this study enabled the reliable detection of chewing strokes in dairy cows using an acceleration sensor. The cleaned algorithm showed a high level of agreement with the manual reference (ICC = 0.91).

Similar approaches have been reported in previous studies using different sensor technologies. For example, pressure-based systems such as RumiWatch (ITIN + HOCH GmbH, Liestal, Switzerland) have demonstrated reliable detection (up to r_s_ = 0.96) of chewing behavior [26]. Accelerometer- and acoustic-based methods have already been successfully applied for detecting chewing activity [18,22], and pressure sensors have shown good agreement with visual observations [19]. Accelerometer-based approaches combined with machine learning have also shown high accuracy in detecting jaw movement activities, reaching classification accuracies of up to 99% [27].

These findings indicate that sensor-based detection of chewing activity is generally feasible, and the results of the present study are in line with previously reported approaches. However, direct comparison between studies remains limited due to differences in sensor placement, signal processing methods, and evaluation metrics.

In the present study, the visually counted chewing strokes per rumination event (median: 66.0) corresponded to approximately 74.3 chewing strokes per minute, based on the mean duration of the analyzed rumination events (53.3 s). These values were therefore near the range of reference values reported in the literature, where approximately 60–70 chewing movements per minute are described [28]. The cleaned version of the algorithm showed a good agreement with the manually counted values from video analysis. At the same time, several aspects became evident that require further development for practical application.

The currently implemented algorithm was based on the detection of individual acceleration peaks in the sensor signal. While this method was generally functional, it has limitations. Especially during regurgitations, sinusoidal movement patterns also occur regularly, which cannot be clearly distinguished from actual chewing events using simple peak detection. As a result, the raw version of the algorithm occasionally produced miscounts, particularly when movements outside actual rumination phases—such as general head movements or regurgitations—were erroneously interpreted as chewing strokes. The cleaned algorithm substantially mitigated this issue by incorporating a delay of approximately five seconds in each regurgitation before initiating the count. This approach corresponds to the observed animal behavior, where a short pause of about 5 s typically occurred between a regurgitation and the resumption of chewing. In accordance with our study, Andriamandroso et al. [15] also concluded that a pre-processing of signals may be required to eliminate existing noises around the animal or during the movement.

For the algorithm to function reliably, the rumination phase must be clearly defined and accurately detected. Without this constraint, there is currently a risk that unrelated movements may be included in the count. The sensor developed by Gouna GmbH (Schönwalde-Glien, Germany) includes a validated system for rumination phase detection, which was not activated in the present study. For future applications, parallel use of this feature is strongly recommended to systematically avoid false detections.

Further development through targeted pattern recognition, for example, using machine learning, also appears promising. Initial observations in this study indicate that characteristic signal patterns emerge during rumination phases. These patterns could serve as a foundation for AI-assisted classification. Of particular note is the consistency of these recurring patterns, which were observed both within individual animals and across different animals. This regularity underscores the potential of the sensor system. Combined with a robust algorithm and the existing rumination phase detection, the system provides a basis for the automated monitoring of chewing behavior. This would make the sensor a valuable tool not only in scientific research but also in practical herd management. Recent reviews have highlighted the increasing importance of accelerometer-based monitoring systems in precision livestock farming and the growing use of machine-learning approaches for automated behaviour classification. Although the present study focused on a transparent rule-based peak detection approach, future developments may combine sensor-based chewing detection with machine-learning methods to improve robustness under more variable field conditions and enable fully automated classification of rumination behaviour [23,29,30,31].

The study was conducted under standardized feeding conditions. How different feed structures—such as a higher proportion of fiber or altered particle lengths—affect the sensor signal and detection accuracy remains unknown. Since ration composition has a direct impact on rumination behavior, this aspect will be specifically addressed in future studies. A comprehensive evaluation under practical, variable feeding conditions is essential to ensure the system’s transferability and reliability in daily herd management.

## 5. Conclusions

The results of this study indicate that chewing strokes can be reliably detected using an acceleration sensor mounted on the animal’s nose. The system was able to count chewing sequences with high accuracy compared to the reference method.

For practical application, however, it is essential that the counting process is linked to detected rumination phases. The rumination phase detection feature integrated into the sensor was not activated in this study but should be enabled in future applications and combined with the detection algorithm. In addition, it would be advisable to expand the current approach by incorporating pattern recognition methods to more reliably exclude movements unrelated to rumination. Since the tests were carried out under standardized feeding conditions, it remains unclear how different feed structures may influence the sensor signal. Future studies should address this aspect to assess the systems transferability under variable practical conditions. Overall, the sensor system provides a promising foundation for the automated detection of chewing activity and offers potential for future use in both research and practical herd management.

## Data Availability

The data presented in this study are available upon request from the corresponding authors.
